# Germinated brown rice combined with *Lactobacillus acidophilus* and *Bifidobacterium animalis* subsp. *lactis* inhibits colorectal carcinogenesis in rats

**DOI:** 10.1002/fsn3.864

**Published:** 2018-11-05

**Authors:** Pao‐Ying Lin, Sing‐Chung Li, Hui‐Pu Lin, Chun‐Kuang Shih

**Affiliations:** ^1^ Division of Gastroenterology and Hepatology Department of Internal Medicine Taipei Medical University Hospital Taipei Taiwan; ^2^ Division of Gastroenterology and Hepatology Department of Internal Medicine School of Medicine College of Medicine Taipei Medical University Taipei Taiwan; ^3^ School of Nutrition and Health Sciences College of Nutrition Taipei Medical University Taipei Taiwan; ^4^ School of Food Safety College of Nutrition Taipei Medical University Taipei Taiwan; ^5^ Master Program in Food Safety College of Nutrition Taipei Medical University Taipei Taiwan

**Keywords:** apoptosis, colorectal cancer, germinated brown rice, probiotics, synbiotics

## Abstract

Colorectal cancer is a common cancer strongly associated with diet. Certain probiotics and prebiotics possess an inhibitory activity against colorectal cancer, while synbiotics may be more effective in preventing this cancer than either prebiotics or probiotics alone. Germinated brown rice (GBR) is considered as a candidate prebiotics with anticancer potential. However, the effect of GBR combined with probiotics on colorectal cancer is not clear. The present study investigated the preventive effect of combination of GBR and *Lactobacillus acidophilus*,* Bifidobacterium animalis* subsp*. lactis*, or both on colorectal carcinogenesis and the possible mechanism in rats treated with 1,2‐dimethylhydrazine (DMH) and dextran sulfate sodium (DSS). DMH/DSS treatment induced preneoplastic aberrant crypt foci (ACF) and mucin‐depleted foci (MDF), reduced superoxide dismutase (SOD) activity, increased anti‐apoptotic Bcl‐2 expression, and decreased the expression of pro‐apoptotic p53, Bax, and caspase‐3 in the colon. Germinated brown rice alone or combined with probiotics inhibited the formation of MDF in the middle colon, enhanced the colonic expression of p53 and Bax, and increased the ratio of Bax/Bcl‐2. Combined treatment of GBR and probiotics inhibited the formation of ACF‐producing sialomucin (SIM‐ACF) and recovered the activity of SOD in the colon. Combination of GBR and *L. acidophilus* further increased caspase‐3 expression and decreased Bcl‐2 expression. These findings suggest that GBR combined with *L. acidophilus* and/or *B. animalis* subsp*. lactis* may inhibit colorectal carcinogenesis by enhancing antioxidative capacity and inducing apoptosis. This synbiotics may be a potential functional food or chemopreventive agent for controlling colorectal cancer.

## 1 INTRODUCTION

Colorectal cancer is the third most common cancer in male population and the second in female population (Torre et al., 2015). In Taiwan, the incidence rates of colorectal cancer rank the first in males and the second in females. These data show that colorectal cancer is a critical health problem in the world. The increased occurrence of colorectal cancer in several countries may reflect the increase in risk factors of this cancer, such as unhealthy diet (Center, Jemal, Smith, & Ward, 2009). In addition, intestinal microbial dysbiosis, the change in population size of certain bacterial species, also plays an important role in colorectal carcinogenesis (So, Law, Law, & Chan, 2016). Some researchers have indicated that modulation of gut microbiota positively affects the interaction between microbiota and immune system and may be beneficial in suppressing colorectal carcinogenesis (Ambalam, Raman, Purama, & Doble, 2016).

According to the definition by FAO/WHO, probiotics are live microorganisms which when administered in adequate amounts confer a health benefit on the host (FAO/WHO, 2006). The majority of probiotics belong to the genera *Lactobacillus* and *Bifidobacterium*, such as *Lactobacillus acidophilus* and *Bifidobacterium animalis* subsp*. lactis*. Several evidences have showed that certain probiotics exert a chemopreventive effect on colorectal cancer through diverse mechanisms (Kahouli, Tomaro‐Duchesneau, & Prakash, 2013). Prebiotics are defined as substrates that are selectively utilized by host microorganisms conferring a health benefit (Gibson et al., 2017). Fructooligosaccharide is one of the most reported prebiotics and is generally recognized as safe. Other prebiotics include galactooligosaccharide, xylooligosaccharide, resistant starch, and so on (Raman et al., 2013). However, only limited studies have investigated the prebiotic potential of complex grains and their products.

Synbiotics mean the combination of probiotics and prebiotics. Emerging data suggest synbiotics as a more effective strategy in the prevention of colorectal cancer than either probiotics or prebiotics alone (Chong, 2014). Common synbiotic combinations for the prevention of colorectal cancer include “*L. acidophilus* plus resistant starch” (Le Leu, Hu, Brown, Woodman, & Young, 2009; Le Leu et al., 2005), “*B. lactis* plus oligofructose/inulin” (Dias et al., 2010), and “*Lactobacillus rhamnosus* GG (LGG) and *B. lactis* Bb12 plus inulin” (Verma & Shukla, 2014). These synbiotics inhibit colorectal carcinogenesis by attenuating oxidative stress, reducing cell proliferation, or inducing apoptosis. However, certain food ingredients with assumed prebiotic potential seem not to be good fermentation substrates for the growth of certain probiotics. For example, some probiotic *Lactobacillus* strains without starch‐degrading activity were not apparently sustained by resistant starch, so they might show a low pro‐apoptotic activity (Le Leu et al., 2005). It suggests the need for development of a novel prebiotic component for the better growth of certain probiotics and their synergistically chemopreventive effect against colorectal cancer.

Whole grains and germinated grains contain soluble dietary fiber, nondigestible oligosaccharides, and resistant starch and thus have been suggested to fulfill the prebiotic concept and to be candidate prebiotics (Bindels, Delzenne, Cani, & Walter, 2015; Broekaert et al., 2011; Hubner & Arendt, 2013) . In recent years, the health‐promoting activity of germinated grains is gaining high interest as a functional food applied to reduce the risk of some chronic diseases, including colorectal cancer (Nelson, Stojanovska, Vasiljevic, & Mathai, 2013). The preliminary study in our laboratory demonstrated that germinated brown rice (GBR) inhibited the development of preneoplastic lesions of colorectal cancer in a carcinogen‐induced animal model (A.‐C. Kuo, C.‐K. Shih, unpublished data). However, the detailed mechanism of action remained to be elucidated. The present study was designed to investigate the preventive effect of combination of GBR with *L. acidophilus* and/or *B. animalis* subsp*. lactis*, two well‐known probiotics with anticancer activity, on colorectal carcinogenesis and the associated mechanism.

## 2 MATERIALS AND METHODS

### 2.1 Materials

1,2‐Dimethylhydrazine dihydrochloride (DMH), alcian blue, dextran sulfate sodium salt (DSS, from *Leuconostoc* spp. average mol wt >500,000), neutral‐buffered formalin solution, and methylene blue were purchased from Sigma‐Aldrich, Inc. (St. Louis, MO, USA). *L. acidophilus* LA5 and *B. animalis* subsp*. lactis* BB‐12 were purchased from Chr. Hansen Holding A/S (Horsholm, Denmark).

### 2.2 GBR preparation

Germinated brown rice used in the present study was obtained from Asia Rice Biotech, Inc. (Taipei, Taiwan). Brown rice (*Oryza sativa*, Taikeng No. 9) was germinated at 37 °C for 22 hr to obtain GBR. Raw GBR was mixed with water and cooked. After cooling treatment, the cooked GBR was frozen‐dried, ground, and screened.

### 2.3 Experimental design

This protocol for animal study was approved by the Institutional Animal Care and Use Committee of Taipei Medical University. Sixty‐six male F344 rats were from the National Laboratory Animal Center (Taipei, Taiwan) and housed in plastic cages in a room maintained at 21 °C with a 12‐hr light–dark cycle. They were divided into six groups. Rats in groups B and D were fed AIN‐93G diet. Others were fed modified AIN‐93G diets containing 10% GBR alone (group G) or combined with *L. acidophilus* (5 × 10^7^ c.f.u./g, group GA), *B. animalis* subsp*. lactis* (5 × 10^7^ c.f.u./g, group GB), and both strains (2.5 × 10^7^ c.f.u./g for each strain, group GAB), respectively. One week after the beginning of experimental diet, all rats except those in group B received DMH (40 mg/kg body weight, i.p.) three times during a week and DSS (2% in drinking water) for 1 week to induce colorectal carcinogenesis. Body weight and food intake were recorded weekly. All rats were sacrificed after 10 weeks of feeding, and colons were collected for analysis.

### 2.4 Assay of aberrant crypt foci (ACF)

Aberrant crypt foci were assessed by an established method (Bird, 1987) and described in our previous study (Li, Chou, & Shih, 2011). Briefly, each colon was cut into three equal‐length sections and fixed between filter papers in formalin solution for 1 days. The fixed colons were stained with methylene blue solution and examined for ACF using a light microscope (Nikon Corp., Tokyo, Japan). The area of the colon was calculated by NIS‐Elements microscope imaging software (Nikon Corp.). The location of each ACF, the number of ACF in each colon section, and the number of aberrant crypt (AC) in each ACF were recorded. Data of ACF and AC were presented as numbers/cm^2^.

### 2.5 Assay of mucin‐producing ACF and mucin‐depleted foci (MDF)

Mucin‐producing ACF and MDF were identified according to the established methods of Jenab, Chen and Thompson (2001)) and Caderni et al. (Caderni et al., 2003), respectively, as described in our previous study (Li et al., 2011). Briefly, the methylene blue‐stained colon was faded with 70% ethanol and then stained using high‐iron diamine alcian blue (HIDAB) method. Firstly, each colon section was stained with high‐iron diamine solution for 30 min and rinsed in distilled water. Secondly, the colon section was stained with 1% alcian blue solution (in 3% acetic acid) for 15 min, rinsed in 80% ethanol followed by distilled water, and finally examined under a light microscope (Nikon Corp.). Brown and blue staining by HIDAB indicated sulfomucin (SUM) and SIM secretion, respectively. SUM‐ACF and SIM‐ACF were defined as ACF with more than 85% SUM‐ and SIM‐stained area, respectively. ACF stained with a smaller percentage of these two mucins were defined as mixed‐type ACF (MIX‐ACF). Furthermore, those with very little or no production of mucins were defined as MDF. The area of the colon was calculated using NIS‐Elements microscope imaging software (Nikon Corp.). Data of mucin‐producing ACF and MDF were presented as numbers/cm^2^.

### 2.6 Measurement of antioxidative enzyme activity

The assay of superoxide dismutase (SOD) activity was performed using Superoxide Dismutase Assay Kit (Cayman Chemical, Ann Arbor, MI, USA). Colonic mucosa was homogenized in five volumes of buffer (1 mM EDTA, 210 mM mannitol, 70 mM sucrose, and 50 mM phosphate buffer, at pH 7.4). The commercial kit was used according to the manufacturer's protocol.

### 2.7 Measurement of apoptosis‐related protein expression

Colonic mucosa were homogenized in modified RIPA buffer (0.5 M Tris–HCl at pH 7.4, 1.5 M sodium chloride, 2.5% deoxycholic acid, 10% NP‐40, and 10 mM EDTA) and 10% protease inhibitor cocktail. The homogenates were centrifuged at 10,000 *g* at 4°C for 15 min, and the supernatants were collected. The protein concentration of the supernatants was determined using the Bio‐Rad Bradford assay (Bio‐Rad, Hercules, CA, USA). Protein samples were separated on 10% sodium dodecyl sulfate (SDS)–polyacrylamide gels and transferred into polyvinylidene difluoride membranes. The membranes were blocked with 5% bovine serum albumin in Tris‐buffered saline containing 1% Tween 20 (TBST) and incubated with p53 (1:1,000 dilution; GeneTex Inc., San Antonio, TX, USA), caspases‐3 (1:1,000 dilution; GeneTex Inc.), Bax (1:1,000 dilution; Cell Signaling Technology, Beverly, MA, USA), or Bcl‐2 (1:1,000 dilution; Cell Signaling Technology) polyclonal antibodies at 4 °C overnight. After 16 hr of incubation at 4 °C and washing with TBST, membranes were incubated with anti‐rabbit or anti‐mouse horseradish peroxidase‐conjugated secondary antibodies for 1 hr. After washing with TBST, the blots were visualized by chemiluminescence reagents and detected with a chemiluminometer (BioSpectrum AC Imaging System; Ultra‐Violet Products, Upland, CA, USA). The blot intensity was quantified by Bio‐Rad Quantity One software (Bio‐Rad Laboratories).

### 2.8 Statistical analysis

All data are expressed as means and standard deviations (*SD*). The difference between experimental groups was assessed by one‐way analysis of variance (ANOVA) followed by Duncan's multiple range test using Statistics Analysis System (SAS Institute, Cary, NC, USA). The *p* values lower than 0.05 were considered statistically significant.

## 3 RESULTS

### 3.1 Body weight and food intake

There were no significant differences in initial body weight, weight gain, food intake, and food efficiency among groups (data not shown).

### 3.2 ACF in the colon

As shown in Table 1, all rats treated with DMH/DSS developed preneoplastic ACF in the colon. Group GA had a significantly lower number of ACF containing one crypt than did group D (*p* < 0.05). There were no significant differences in the numbers of ACF containing more crypts and total ACF among groups.

**Table 1:** Effects of germinated brown rice and synbiotics on DMH/DSS‐induced ACF (number/cm^2^) according to the various size of crypts in the colon of male F344 rats^a^^,^ b

Group c	Incidence d	ACF with			Small ACF (≦3 ACs)	Large ACF (≧4 ACs)	Total ACF
		1 AC	2 ACs	3 ACs			
D	100% (9/9)	2.2 ± 0.8^A^	3.3 ± 0.9	1.6 ± 0.5	7.0 ± 1.8	0.7 ± 0.4	7.8 ± 2.1
G	100% (12/12)	1.7 ± 0.8^A,B^	2.9 ± 1.1	1.6 ± 0.7	6.1 ± 2.3	0.7 ± 0.4	6.9 ± 2.6
GA	100% (12/12)	1.1 ± 0.6^B^	2.6 ± 1.2	1.6 ± 1.1	5.3 ± 2.6	0.8 ± 0.5	6.1 ± 2.9
GB	100% (12/12)	1.6 ± 0.6^A,B^	2.9 ± 0.7	1.5 ± 0.4	6.0 ± 1.4	0.6 ± 0.4	6.6 ± 1.5
GAB	100% (12/12)	1.6 ± 0.8^A,B^	2.9 ± 1.3	2.0 ± 0.7	6.6 ± 2.4	1.0 ± 0.3	7.5 ± 2.5

^a^All values are mean ± *SD* (*n* = 9–12). ^b^Values with the same letter in a column are not significantly different from one another as determined by ANOVA and Duncan's multiple range test, *p* < 0.05. ^c^All rats were administered with DMH/DSS. D, AIN‐93G diet; G, AIN‐93G containing 10% germinated brown rice; GA, AIN‐93G containing 10% GBR + 5 × 107 c.f.u./g *L. acidophilus*; GB, AIN‐93G containing 10% GBR + 5 × 107 c.f.u./g *B. animalis* subsp. *lactis*; GAB, AIN‐93G containing 10% GBR + 2.5 × 107 c.f.u./g *L. acidophilus* + 2.5 × 107 c.f.u./g diet *B. animalis* subsp. *lactis*. ^d^Number of rats with ACF/total rats.

### 3.3 Mucin secretion by ACF

The majority of ACF in the colon secreted SUM (Table**** 2). There were no significant differences in the numbers of ACF producing either SUM or mixed SUM and SIM among groups. However, groups GA, GB, and GAB had significantly lower numbers of ACF producing SIM than did group D (*p* < 0.05).

**Table 2:** Effects of germinated brown rice and synbiotics on the number of DMH/DSS‐induced ACF according to the type of mucin produced by foci in the total colon of male F344 rats a ^,^ b

Group^c^	Number of ACF producing (number/cm^2^)		
	SUM	MIX	SIM
D	5.9 ± 1.8	0.9 ± 0.4	0.9 ± 0.6^A^
G	4.9 ± 1.9	1.0 ± 0.5	0.9 ± 0.7^A^
GA	4.7 ± 2.3	0.9 ± 0.5	0.5 ± 0.4^B^
GB	4.9 ± 1.4	1.2 ± 0.5	0.4 ± 0.3^B^
GAB	6.1 ± 2.4	1.1 ± 0.6	0.3 ± 0.2^B^

Notes

SIM: sialomucin; SUM: sulfomucin; MIX: mixed sulfomucin and sialomucin.

^a^All values are mean ± *SD* (*n* = 9–12). ^b^Values with the same letter in a column are not significantly different from one another as determined by Duncan's multiple range test, *p* < 0.05. ^c^All rats were administered with DMH/DSS. D, AIN‐93G diet; G, AIN‐93G containing 10% germinated brown rice; GA, AIN‐93G containing 10% GBR + 5 × 107 c.f.u./g *L. acidophilus*; GB, AIN‐93G containing 10% GBR + 5 × 107 c.f.u./g *B. animalis* subsp. *lactis*; GAB, AIN‐93G containing 10% GBR + 2.5 × 107 c.f.u./g *L. acidophilus* + 2.5 × 107 c.f.u./g *B. animalis* subsp*. lactis*.

### 3.4 MDF in the colon

The incidence of MDF and their distribution in the colon is shown in Table 3. The highest incidence rate (66%) of MDF was observed in group D, although there was no significant difference among groups. MDF mainly developed in the distal colon, while no MDF appeared in the proximal colon. Groups G, GA, GB, and GAB had significantly lower numbers of MDF in the middle colon than did group D (*p* < 0.05).

**Table 3:** Effects of germinated brown rice and synbiotics on the distribution of DMH/DSS‐induced MDF (number/cm^2^) in the colon of male F344 rats a ^,^ b

Group c	Incidence (number of rats with MDF/total rats)	Proximal colon	Middle colon	Distal colon	Total MDF
D	66% (6/9)	0.00 ± 0.00	0.06 ± 0.12^A^	0.12 ± 0.25	0.06 ± 0.08
G	25% (3/12)	0.00 ± 0.00	0.00 ± 0.00^B^	0.12 ± 0.22	0.04 ± 0.08
GA	16% (2/12)	0.00 ± 0.00	0.00 ± 0.00^B^	0.05 ± 0.13	0.02 ± 0.04
GB	33% (4/12)	0.00 ± 0.00	0.00 ± 0.00^B^	0.13 ± 0.21	0.04 ± 0.06
GAB	16% (2/12)	0.00 ± 0.00	0.00 ± 0.00^B^	0.07 ± 0.17	0.02 ± 0.05

^a^All values are mean ± *SD* (*n* = 9–12). ^b^Values with the same letter in a column are not significantly different from one another as determined by ANOVA and Duncan's multiple range test, *p* < 0.05. ^c^All rats were administered with DMH/DSS. D, AIN‐93G diet; G, AIN‐93G containing 10% germinated brown rice; GA, AIN‐93G containing 10% GBR + 5 × 107 c.f.u./g *L. acidophilus*; GB, AIN‐93G containing 10% GBR + 5 × 107 c.f.u./g *B. animalis* subsp. *lactis*; GAB, AIN‐93G containing 10% GBR + 2.5 × 107 c.f.u./g *L. acidophilus* + 2.5 × 107 c.f.u./g *B. animalis* subsp. *lactis*.

### 3.5 Colonic SOD activity

**Figure 1:**
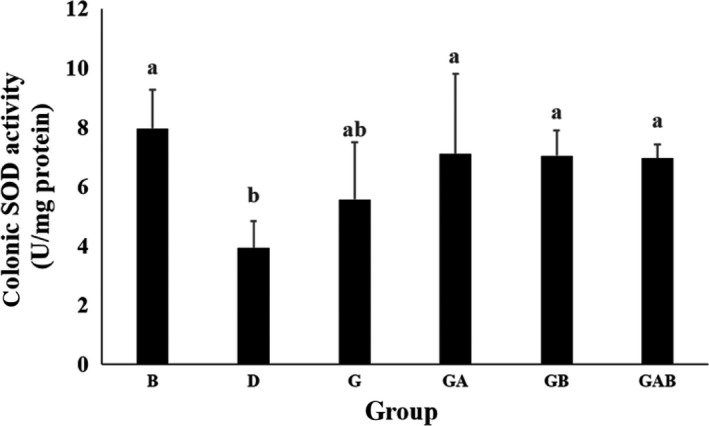
Effect of germinated brown rice and synbiotics on colonic superoxide dismutase activity in male F344 rats. The bars represent mean ± S*D*. All rats except group B were administered with DMH/DSS. B and D, AIN‐93G diet; G, AIN‐93G containing 10% germinated brown rice; Ga, AIN‐93G containing 10% GBR + 5 × 10^7^ c.f.u./g *L. acidophilus*; GB, AIN‐93G containing 10% GBR + 5 × 10^7^ c.f.u./g *B. animalis* subsp. *lactis*; GAB, AIN‐93G containing 10% GBR + 2.5 × 10^7^ c.f.u./g *L. acidophilus* + 2.5 × 10^7^ c.f.u./g *B. animalis* subsp. *lactis*

As shown in Figure 1, DMH/DSS treatment decreased the SOD activity in colonic mucosa. Group D had significantly lower SOD activity than did group B (*p* < 0.05). The SOD activities were significantly elevated in groups GA, GB, and GAB compared with group D (*p* < 0.05).

### 3.6 Apoptosis‐related proteins in the colon

**Figure 2:**
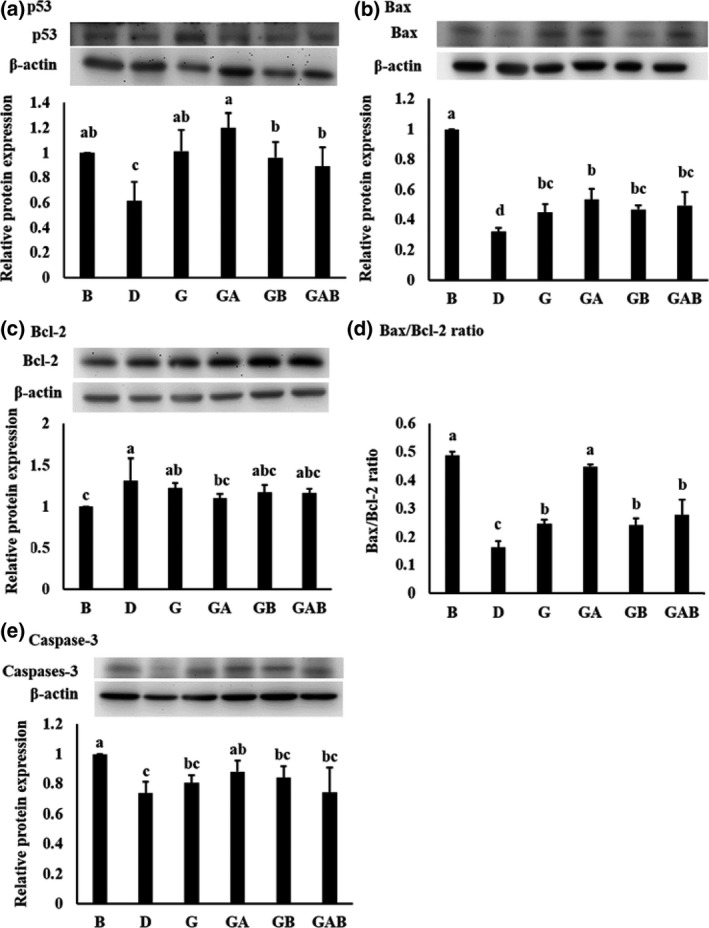
Effect of germinated brown rice and synbiotics on (a) p53, (b) Bax, (c) Bcl‐2, (d) Bax/Bcl‐2 ratio, and (e) caspase‐3 expression of colonic mucosa in male F344 rats. The bars represent mean ± *SD*. All rats except group B were administered with DMH/DSS. B and D, AIN‐93G diet; G, AIN‐93G containing 10% germinated brown rice; GA, AIN‐93G containing 10% GBR + 5 × 10^7^ c.f.u./g *L. acidophilus*; GB, AIN‐93G containing 10% GBR + 5 × 10^7^ c.f.u./g *B. animalis* subsp. *lactis*; GAB, AIN‐93G containing 10% GBR *+* 2.5 × 10^7^ c.f.u./g *L. acidophilus* + 2.5×10^7^ c.f.u./g *B. animalis* subsp. *lactis*

The expressions of apoptosis‐related proteins in colonic mucosa are shown in Figure 2. DMH/DSS treatment decreased the expression of pro‐apoptotic protein and increased the expression of anti‐apoptotic protein. Group D showed significantly lower expressions of p53, Bax, and caspase‐3 (*p* < 0.05) and a significantly higher expression of Bcl‐2 (*p* < 0.05) than did group B. The Bax/Bcl‐2 ratio was also significantly lower in group D than that in group B (*p* < 0.05). In contrast, consumption of GBR and probiotics improved the abnormal expression of apoptosis‐related proteins. The expressions of p53 and Bax as well as Bax/Bcl‐2 ratio were significantly increased in groups G, GA, GB, and GAB compared with group D (*p* < 0.05). Group GA showed significantly higher (*p* < 0.05) caspase‐3 expression and significantly lower (*p* < 0.05) Bcl‐2 expression than did group D.

## 4 DISCUSSION

To our knowledge, this is the first study investigating the preventive effect of combination of GBR and probiotics (*L. acidophilus* and *B. animalis* subsp*. lactis*) on colorectal carcinogenesis. Our data indicate that combination of GBR and probiotics may inhibit preneoplastic lesions and regulate antioxidative enzyme and apoptosis‐related proteins in the colon. These findings suggest that such synbiotic combination may have the potential to serve as a novel chemopreventive agent for colorectal cancer.

Aberrant crypt foci are the early lesions observed in the colon of animals treated with carcinogen and of humans at cancer risk. They are commonly used as a biomarker in chemically induced animal models for studies of colorectal carcinogenesis and its prevention or therapy (Corpet & Taché, 2002). The present study showed that consumption of GBR and *L. acidophilus* (group GA) significantly inhibited the formation of ACF with 1 aberrant crypt, the smallest ACF, although GBR alone (group G) did not have such significant inhibitory effect on ACF. This result suggests that combination of GBR and *L. acidophilus* may intervene in the early stage of colorectal carcinogenesis and that *L. acidophilus* may play a key role in prevention of colorectal cancer. Previous studies have confirmed the anticancer effect of probiotic Lactobacilli strains. Liboredo et al. (2013) demonstrated that consumption of *Lactobacillus delbrueckii* UFVH2b20 resulted in a significant reduction in the number of colonic ACF, especially small ACF, in DMH‐induced mice. It supports the inhibitory activity of some Lactobacilli strains in early colorectal carcinogenesis as observed in our study. Gamallat et al. (2016) reported that LGG treatment significantly reduced DMH‐induced tumor incidence, multiplicity, and volume in the colon of rats. The antitumor effect was related to induction of apoptosis and suppression of inflammation. Chen et al. (2012) used an animal model of segmental orthotopic colon cancer in which mice were implanted with CT‐26 murine colon adenocarcinoma cells. They found that *L. acidophilus* inhibited the growth of tumor and induced the apoptosis of tumor cells. Soltan Dallal et al. (2015) indicated that either supernatants or extracts of two Lactobacillus species (*L. acidophilus* and *L. casei*) suppressed the malignant phenotype of Caco‐2 cells by decreasing cell proliferation, migration, and invasion and increasing cell apoptosis. These studies suggest that some Lactobacilli strains also exert an anticancer effect in the relatively late (promotion and progression) stage of colorectal carcinogenesis and that enhancement of cell apoptosis may be an important mechanism implicated in the suppression of colorectal cancer.

The present study did not observe a significant inhibitory effect on ACF formation in rats fed the combination of GBR and *B. animalis* subsp*. lactis*. Some studies have reported the anticancer effect of probiotic Bifidobacteria strains. Altonsy, Andrews, and Tuohy (2010) found that *B. lactis* Bb12 provided protection against Caco‐2 human epithelial colorectal adenocarcinoma cells by inducing apoptosis via the mitochondrial route. Liboredo et al. (2013) showed that consumption of *B. animalis* var. *lactis* Bb12 significantly reduced the number of colonic ACF, especially large ACF, in mice treated with DMH. In contrast, *B. lactis* alone had neither promoting effect on butyrate production nor inhibitory effect on colonic tumors in carcinogen azoxymethane‐induced rats, although there was still a protective effect against colonic tumors in rats ingested both *B. lactis* and prebiotic‐resistant starch (Le Leu et al., 2009). Another study also found the combination of *B. lactis* and prebiotic oligofructose/inulin could suppress the development of both classical and more advanced ACF (MDF) in DMH‐treated rats (Dias et al., 2010). These studies suggest the importance of suitable substrates (prebiotics) for the fermentation and the anticancer function of probiotic Bifidobacteria strains, especially for suppression of advanced colonic lesions.

Aberrant crypt foci are heterogeneous lesions. Most ACF are hyperplastic and considered not to convert into tumors; however, dysplastic ACF are generally considered as preneoplastic lesions (Kim, Guo, O'sullivan, Gallaher, & Turesky, 2016). Researchers have proposed some classifications of ACF in order to distinguish their malignancy. Classification based on mucin production in ACF is one of the common methods. Mucins are important molecular barriers existed on the surface of colon, protecting the epithelium against mechanical and chemical damages. Goblet cells are secretory cells contributing to the release of intestinal mucins, such as Muc2, the main component of the intestinal mucus barrier (Moulahoum, Nagy, Djerdjouri, & Clichici, 2018). During colorectal carcinogenesis, there are emerging changes in the structure and the amount of mucins, causing dysfunction of mucus barrier and abnormal cell signal transduction (Milosevic et al., 2015). The alteration of mucins is presented primarily by excessive SIM secretion in patients with colorectal cancer and a significant reduction in SUM (Milosevic et al., 2015). It has been shown that SUM secretion is negatively correlated to proliferative index, while SIM production is positively correlated to proliferative activity (Milosevic et al., 2015). An increase in SIM secretion and a decrease in SUM secretion have been observed during colorectal carcinogenesis in both humans and animals. In rats, normal colonic crypts and hyperplastic ACF are stained for SUM, whereas dysplastic ACF are stained for SIM, and the most dysplastic crypts (MDF) do not show any mucin staining (Kim et al., 2016). The above findings confirm that SIM‐ACF and MDF are advanced preneoplastic lesions during colorectal carcinogenesis.

In the present study, though the total numbers of ACF were not affected by GBR and probiotics, all combined treatments (groups GA, GB, and GAB) significantly reduced the numbers of SIM‐ACF in whole colon and MDF in the middle colon. GBR alone (group G) also reduced the number of MDF in the middle colon. These results suggest that GBR and probiotics used in this study may modulate colonic secretion of mucins and their alterations during colorectal carcinogenesis. A previous study indicated that the presence of fecal residues rich in short‐chain fatty acids (SCFAs) in the colon might maintain the energy supply and the mucin synthesis (Martinez et al., 2010). It confirms the ability of colonic epithelium for mucin synthesis depends on enough SCFAs supply. It is possible that consumption of GBR, a good substrate for certain colonic bacteria, promotes fermentation and SCFAs production in the colon, and thus prevents the depletion of mucins and the formation of MDF. The inhibitory effect of GBR and probiotics on preneoplastic SIM‐ACF and MDF also suggests that GBR is a good substrate for the growth of both *L. acidophilus* and *B. animalis* subsp*. lactis*, so the combination of GBR and these probiotics may maintain the anticancer activity of probiotics as reported in previous studies. In addition, the present study also shows the potential of GBR in prevention of colorectal cancer.

Previous studies have provided several lines of evidence that oxidative stress may increase the risk of gastrointestinal cancer (Law, Waye, & So, 2017). Antioxidative enzymes exert a protective role in the intestinal epithelium (Perez, Talens‐Visconti, Rius‐Perez, Finamor, & Sastre, 2017). In general, exposure to carcinogens reduces the activities of enzymatic and nonenzymatic antioxidants (Moulahoum et al., 2018). For example, DMH treatment decreased the activity of colonic SOD, catalase (CAT), glutathione peroxidase (GPx), and glutathione‐S‐transferase in rats (Walia, Kamal, Dhawan, & Kanwar, 2018). The activity of SOD and CAT decreased significantly after DMH administration, confirming an imbalance of oxidative stress in the colon (Moulahoum et al., 2018). Lactic acid bacteria (LAB) could decrease the activity of carcinogen by eliminating the reactive intermediates and elevating the levels of antioxidative enzymes (Verma & Shukla, 2014). Genetically modified LAB expressing antioxidative enzymes (SOD or CAT) showed an anticancer effect in a rodent model of colorectal cancer (Del Carmen et al., 2017).

Lactobacillus strains possess an antioxidative capacity which may be related to cell metabolites. A surface layer protein (SLP) isolated from *L. acidophilus* NCFM played an important role in the antioxidative capacity of HT‐29 cells induced by H_2_O_2_ (Zhao, Meng, Zhang, Kang, & Lu, 2017). This SLP improved the total antioxidative capacity (T‐AOC) and the activities of CAT and SOD and decreased the level of malondialdehyde (Zhao et al., 2017). In addition, the antioxidative property of exopolysaccharides (EPS) has been assessed in several probiotics such as *Lactococcus lactis* and *Lactobacillus plantarum* which have been reported to promote the production of antioxidative enzymes such as SOD (Deepak et al., 2016). Bifidobacteria strains also show an antioxidative capacity related to EPS. The EPS synthesized by *B. longum* W11 acted as an antioxidant against H_2_O_2_‐induced reactive oxygen species in an in vitro cell model (Inturri et al., 2017).

The present study found that all combinations of GBR and probiotics (groups GA, GB, and GAB) significantly improved the decreased colonic SOD activity induced by DMH/DSS treatment and maintained the activity similar to that of normal rats (group B). Interestingly, the modulatory effect of GBR and probiotics on colonic mucin alteration showed a similar phenomenon that all combinations of GBR and probiotics (groups GA, GB, and GAB) significantly inhibited the formation of SIM‐ACF induced by DMH/DSS. These results suggest that synbiotic combination of GBR and probiotics are more effective in antioxidative activity than GBR given individually. Also, these findings reveal the possibility that enhancement of antioxidative status or reduction of oxidative stress is associated with the suppression of malignant mucin transition in the colon.

The acute apoptotic response to a genotoxic carcinogen (such as DMH) could regulate mutation in the colon and eliminate cells with abnormal DNA that might progress to tumors, so this response might exert a protective effect at the early stage during colorectal carcinogenesis in rats (Le Leu et al., 2005). 1,2‐Dimethylhydrazine treatment led to changes in the protein expressions of certain genes involved in the intrinsic apoptosis pathway such as p53, Bax, Bcl‐2, and caspase‐3 (Walia et al., 2018). A synbiotic combination of *B. lactis* and RS significantly induced the apoptotic response to a genotoxic carcinogen in the distal colon of rats in a short‐term study (Le Leu et al., 2005). RS induced alterations of intestinal microenvironment including acidification of digests, production of SCFAs, and changes in the balance of microbial species, which might create a situation that enabled *B. lactis* to exert a pro‐apoptotic effect (Le Leu et al., 2005). However, the same synbiotic combination used in a long‐term (26 weeks) study showed no significant difference in spontaneous apoptotic cells in treatment groups, although this synbiotics significantly protected against the development of colorectal tumors (Le Leu et al., 2009). These findings suggest that apoptosis is an early event during colorectal carcinogenesis and that anticancer agents with apoptosis‐inducing ability may tend to be effective at the early stage of colorectal carcinogenesis. The present study was a short‐term (10 weeks) study showing that consumption of GBR alone (group G) or combined with probiotics (groups GA, GB, and GAB) increased the protein expression of pro‐apoptotic p53 and Bax and the ratio of Bax/Bcl‐2. In addition, the combination of GBR and *L. acidophilus* (group A) further increased pro‐apoptotic caspase‐3 expression and decreased anti‐apoptotic Bcl‐2 expression. These results suggest that GBR and probiotics may promote cell apoptosis via p53‐mediated pathway during colorectal carcinogenesis. The combination of GBR and *L. acidophilus* may be the most effective synbiotics in facilitating apoptosis.

The effect of GBR on apoptosis induction may be associated with SCFAs produced from GBR by certain colonic bacteria as observed in prebiotic‐involved studies. It has been reported that SCFAs are able to induce epigenetic changes, arrest cell cycle, and increase the expression of pro‐apoptotic genes (Dos Reis et al., 2017). The promoting effect of probiotics on apoptosis may be through SCFA production, immunomodulation, and increased expression of genes and proteins involved in regulation of apoptosis pathway (Dos Reis et al., 2017). Consumption of *L. acidophilus* suppressed the growth of tumor and enhanced the apoptosis of tumor cells in an animal model of segmental orthotopic colon cancer, in which mice were implanted with CT‐26 murine colon adenocarcinoma cells (Chen et al., 2012). There was a lower Bcl‐2 expression in CT‐26 cells isolated from mice pre‐inoculated with *L. acidophilus* compared with those from untreated mice*. *Moreover, the expressions of caspase‐3 and caspase‐9 were higher in *L. acidophilus*‐treated cells compared with those from untreated mice (Chen et al., 2012). *B. lactis* Bb12 induced apoptosis through the mitochondrial pathway including Bax translocation, cytochrome c release, and cleavages of caspase‐9 and caspase‐3 in human colonic carcinoma cells (Altonsy et al., 2010). The impact of synbiotics on apoptosis is a combined effect of both probiotics and prebiotics and usually attributed to the fermentation‐mediated production of luminal SCFAs, especially butyrate (Borowicki et al., 2011). An interaction between butyrate produced via fermentation of prebiotics and immunomodulating properties of probiotics may be another possible explanation (Le Leu et al., 2009). The present study showed that GBR alone and its combination with *L. acidophilus* and/or *B. animalis* subsp*. lactis* may act as an apoptosis promoter through p53‐mediated pathway and may be expected as a potential modulator of colorectal carcinogenesis.

Previous studies found that both supernatants and extracts of *L. acidophilus* decreased cell proliferation and increased apoptosis in colorectal cancer cells (Soltan Dallal et al., 2015). Exopolysaccharides are natural polymers of monosaccharides produced by various microorganisms which are either bound to the cell surface or released into the surrounding environment (Deepak et al., 2016). Lactic acid bacteria is the well‐known bacteria synthesizing EPSs which adhere to the bacterial surface followed by release in surrounding medium (El‐Deeb, Yassin, Al‐Madboly, & El‐Hawiet, 2018). Exopolysaccharides synthesized by some LAB play an important role in colonization of LAB in the intestinal mucosa and show many health benefits such as antitumor, immune‐enhancing, and antioxidative properties (El‐Deeb et al., 2018). The EPS from *L. acidophilus* had antioxidative properties, reduced viability of colon cancer cell lines, downregulated the expression of genes involved in angiogenesis, and upregulated the expression of antiangiogenic genes in HCT15 and Caco‐2 cells (Deepak et al., 2016). A novel EPS purified from *L. acidophilus* exerted an antiproliferative effect on Caco‐2 cells via the apoptotic mechanism in addition to inactivating the inflammatory pathway and stimulating the immune response (El‐Deeb et al., 2018). This EPS increased the ratio of apoptotic cells in sub‐G0/G1 cell cycle phase and upregulated the expression of IκBα, P53, and TGF genes (El‐Deeb et al., 2018). The EPS produced by probiotics may explain, at least partly, the inhibitory effect of probiotics and synbiotics against colorectal carcinogenesis, although further studies are needed to elucidate the exact mechanism.

## 5 CONCLUSION

In summary, the present study shows that combination of GBR and probiotics *L. acidophilus* and/or *B. animalis* subsp*. lactis* may suppress DMH/DSS‐induced colonic preneoplastic lesions via regulating antioxidation and apoptosis in rats. These findings suggest that such synbiotic combination may be a potential functional food or chemopreventive agent for control of colorectal cancer.

## CONFLICT OF INTEREST

The authors declare that there are no conflict of interests.

## ETHICAL APPROVAL

The animal care and experimental procedures were approved by the Institutional Animal Care and Use Committee (IACUC) of Taipei Medical University (Approval No. LAC‐2014‐0118) and conducted according to the guidelines of the Management of Laboratory Animals in Taipei Medical University.

## References

[fsn3864-bib-0001] Altonsy, M. O. , Andrews, S. C. , & Tuohy, K. M. (2010). Differential induction of apoptosis in human colonic carcinoma cells (Caco‐2) by Atopobium, and commensal, probiotic and enteropathogenic bacteria: Mediation by the mitochondrial pathway. International Journal of Food Microbiology, 137(2‐3), 190–203. 10.1016/j.ijfoodmicro.2009.11.015 20036023

[fsn3864-bib-0002] Ambalam, P. , Raman, M. , Purama, R. K. , & Doble, M. (2016). Probiotics, prebiotics and colorectal cancer prevention. Best Practice and Research: Clinical Gastroenterology, 30(1), 119–131. 10.1016/j.bpg.2016.02.009 27048903

[fsn3864-bib-0003] Bindels, L. B. , Delzenne, N. M. , Cani, P. D. , & Walter, J. (2015). Towards a more comprehensive concept for prebiotics. Nature Reviews Gastroenterology and Hepatology, 12(5), 303 10.1038/nrgastro.2015.47 25824997

[fsn3864-bib-0004] Bird, R. P. (1987). Observation and quantification of aberrant crypts in the murine colon treated with a colon carcinogen: Preliminary findings. Cancer Letters, 37(2), 147–151. 10.1016/0304-3835(87)90157-1 3677050

[fsn3864-bib-0005] Borowicki, A. , Michelmann, A. , Stein, K. , Scharlau, D. , Scheu, K. , Obst, U. , & Glei, M. (2011). Fermented wheat aleurone enriched with probiotic strains LGG and Bb12 modulates markers of tumor progression in human colon cells. Nutrition and Cancer, 63(1), 151–160. 10.1080/01635581.2010.516874 21161821

[fsn3864-bib-0006] Broekaert, W. F. , Courtin, C. M. , Verbeke, K. , Van de Wiele, T. , Verstraete, W. , & Delcour, J. A. (2011). Prebiotic and other health‐related effects of cereal‐derived arabinoxylans, arabinoxylan‐oligosaccharides, and xylooligosaccharides. Critical Reviews in Food Science and Nutrition, 51(2), 178–194. 10.1080/10408390903044768 21328111

[fsn3864-bib-0007] Caderni, G. , Femia, A. P. , Giannini, A. , Favuzza, A. , Luceri, C. , Salvadori, M. , & Dolara, P. (2003). Identification of mucin‐depleted foci in the unsectioned colon of azoxymethane‐treated rats: Correlation with carcinogenesis. Cancer Research, 63(10), 2388–2392. 12750256

[fsn3864-bib-0008] Center, M. M. , Jemal, A. , Smith, R. A. , & Ward, E. (2009). Worldwide variations in colorectal cancer. CA: A Cancer Journal for Clinicians, 59(6), 366–378. 10.3322/caac.20038 19897840

[fsn3864-bib-0009] Chen, C.‐C. , Lin, W.‐C. , Kong, M.‐S. , Shi, H. N. , Walker, W. A. , Lin, C.‐Y. , … Lin, T.‐Y. (2012). Oral inoculation of probiotics Lactobacillus acidophilus NCFM suppresses tumour growth both in segmental orthotopic colon cancer and extra‐intestinal tissue. British Journal of Nutrition, 107(11), 1623–1634. 10.1017/S0007114511004934 21992995

[fsn3864-bib-0010] Chong, E. S. L. (2014). A potential role of probiotics in colorectal cancer prevention: Review of possible mechanisms of action. World Journal of Microbiology and Biotechnology, 30(2), 351–374. 10.1007/s11274-013-1499-6 24068536

[fsn3864-bib-0011] Corpet, D. E. , & Taché, S. (2002). Most effective colon cancer chemopreventive agents in rats: A systematic review of aberrant crypt foci and tumor data, ranked by potency. Nutrition and Cancer, 43(1), 1–21. 10.1207/S15327914NC431_1 12467130 PMC2536533

[fsn3864-bib-0012] Deepak, V. , Ramachandran, S. , Balahmar, R. M. , Pandian, S. R. K. , Sivasubramaniam, S. D. , Nellaiah, H. , & Sundar, K. (2016). In vitro evaluation of anticancer properties of exopolysaccharides from *Lactobacillus acidophilus* in colon cancer cell lines. In Vitro Cellular and Developmental Biology‐Animal, 52(2), 163–173. 10.1007/s11626-015-9970-3 26659393

[fsn3864-bib-0013] Del Carmen, S. , de Moreno de LeBlanc, A. , Levit, R. , Azevedo, V. , Langella, P. , Bermudez‐Humaran, L. G. , & LeBlanc, J. G. (2017). Anti‐cancer effect of lactic acid bacteria expressing antioxidant enzymes or IL‐10 in a colorectal cancer mouse model. International Immunopharmacology, 42, 122–129. 10.1016/j.intimp.2016.11.017 27912148

[fsn3864-bib-0014] Dias, M. C. , Vieiralves, N. F. , Gomes, M. I. , Salvadori, D. M. , Rodrigues, M. A. , & Barbisan, L. F. (2010). Effects of lycopene, synbiotic and their association on early biomarkers of rat colon carcinogenesis. Food and Chemical Toxicology, 48(3), 772–780. 10.1016/j.fct.2009.12.003 20026158

[fsn3864-bib-0015] Dos Reis, S. A. , da Conceicao, L. L. , Siqueira, N. P. , Rosa, D. D. , da Silva, L. L. , & Peluzio, M. D. (2017). Review of the mechanisms of probiotic actions in the prevention of colorectal cancer. Nutrition Reviews, 37, 1–19. 10.1016/j.nutres.2016.11.009 28215310

[fsn3864-bib-0016] El‐Deeb, N. M. , Yassin, A. M. , Al‐Madboly, L. A. , & El‐Hawiet, A. (2018). A novel purified *Lactobacillus acidophilus* 20079 exopolysaccharide, LA‐EPS‐20079, molecularly regulates both apoptotic and NF‐κB inflammatory pathways in human colon cancer. Microbial Cell Factories, 17(1), 29 10.1186/s12934-018-0877-z 29466981 PMC5820793

[fsn3864-bib-0017] FAO/WHO (2006). Probiotics in food. Health and nutritional properties and guidelines for evaluation. FAO Food and Nutrition Paper 85

[fsn3864-bib-0018] Gamallat, Y. , Meyiah, A. , Kuugbee, E. D. , Hago, A. M. , Chiwala, G. , Awadasseid, A. , … Luo, F. (2016). *Lactobacillus rhamnosus* induced epithelial cell apoptosis, ameliorates inflammation and prevents colon cancer development in an animal model. Biomedicine and Pharmacotherapy, 83, 536–541. 10.1016/j.biopha.2016.07.001 27447122

[fsn3864-bib-0019] Gibson, GR , Hutkins, R , Sanders, ME , Prescott, SL , Reimer, RA , Salminen, SJ , … Reid, G. (2017). Expert consensus document: The International Scientific Association for Probiotics and Prebiotics (ISAPP) consensus statement on the definition and scope of prebiotics. Nature Reviews Gastroenterology and Hepatology, 14(8), 491–502. 10.1038/nrgastro.2017.75 28611480

[fsn3864-bib-0020] Hubner, F. , & Arendt, E. K. (2013). Germination of cereal grains as a way to improve the nutritional value: A review. Critical Reviews in Food Science and Nutrition, 53(8), 853–861. 10.1080/10408398.2011.562060 23768147

[fsn3864-bib-0021] Inturri, R. , Molinaro, A. , Di Lorenzo, F. , Blandino, G. , Tomasello, B. , Hidalgo‐Cantabrana, C. , … Ruas‐Madiedo, P. (2017). Chemical and biological properties of the novel exopolysaccharide produced by a probiotic strain of *Bifidobacterium longum* . Carbohydrate Polymers, 174, 1172–1180. 10.1016/j.carbpol.2017.07.039 28821042

[fsn3864-bib-0022] Jenab, M. , Chen, J. , & Thompson, L. U. (2001). Sialomucin production in aberrant crypt foci relates to degree of dysplasia and rate of cell proliferation. Cancer Letters, 165(1), 19–25. 10.1016/S0304-3835(00)00706-0 11248414

[fsn3864-bib-0023] Kahouli, I. , Tomaro‐Duchesneau, C. , & Prakash, S. (2013). Probiotics in colorectal cancer (CRC) with emphasis on mechanisms of action and current perspectives. Journal of Medical Microbiology, 62(Pt 8), 1107–1123. 10.1099/jmm.0.048975-0 23558140

[fsn3864-bib-0024] Kim, S. , Guo, J. , O'sullivan, M. G. , Gallaher, D. D. , & Turesky, R. J. (2016). Comparative DNA adduct formation and induction of colonic aberrant crypt foci in mice exposed to 2‐amino‐9H‐pyrido [2, 3‐b] indole, 2‐amino‐3, 4‐dimethylimidazo [4, 5‐f] quinoline, and azoxymethane. Environmental and Molecular Mutagenesis, 57(2), 125–136. 10.1002/em.21993 26734915 PMC4752904

[fsn3864-bib-0025] Law, B. M. , Waye, M. M. , & So, W. K. (2017). Hypotheses on the potential of rice bran intake to prevent gastrointestinal cancer through the modulation of oxidative stress. International Journal of Molecular Sciences, 18(7), 1352 10.3390/ijms18071352 PMC5535845 28672811

[fsn3864-bib-0026] Le Leu, R. K. , Brown, I. L. , Hu, Y. , Bird, A. R. , Jackson, M. , Esterman, A. , & Young, G. P. (2005). A synbiotic combination of resistant starch and *Bifidobacterium lactis* facilitates apoptotic deletion of carcinogen‐damaged cells in rat colon. Journal of Nutrition, 135(5), 996–1001. 10.1093/jn/135.5.996 15867271

[fsn3864-bib-0027] Le Leu, R. K. , Hu, Y. , Brown, I. L. , Woodman, R. J. , & Young, G. P. (2009). Synbiotic intervention of Bifidobacterium lactis and resistant starch protects against colorectal cancer development in rats. Carcinogenesis, 31(2), 246–251. 10.1093/carcin/bgp197 19696163

[fsn3864-bib-0028] Li, S.‐C. , Chou, T.‐C. , & Shih, C.‐K. (2011). Effects of brown rice, rice bran, and polished rice on colon carcinogenesis in rats. Food Research International, 44(1), 209–216. 10.1016/j.foodres.2010.10.034

[fsn3864-bib-0029] Liboredo, J. C. , Anastácio, L. R. , Pelúzio, M. d. C. G. , Valente, F. X. , Penido, L. C. P. , Nicoli, J. R. , & Correia, M. I. T. D. (2013). Effect of probiotics on the development of dimethylhydrazine‐induced preneoplastic lesions in the mice colon. Acta Cirurgica Brasileira, 28(5), 367–372. 10.1590/s0102-86502013000500008 23702939

[fsn3864-bib-0030] Martinez, C. A. , Nonose, R. , Spadari, A. P. , Maximo, F. R. , Priolli, D. G. , Pereira, J. A. , & Margarido, N. F. (2010). Quantification by computerized morphometry of tissue levels of sulfomucins and sialomucins in diversion colitis in rats. Acta Cirurgica Brasileira, 25(3), 231–240. https://doi.org/; 10.1590/S0102-86502010000300004 20498935

[fsn3864-bib-0031] Milosevic, V. , Vukmirovic, F. , Zindovic, M. , Krstic, M. , Milenkovic, S. , & Jancic, S. (2015). Interplay between expression of leptin receptors and mucin histochemical aberrations in colorectal adenocarcinoma. Romanian Journal of Morphology and Embryology, 56(2 Suppl), 709–716. 26429163

[fsn3864-bib-0032] Moulahoum, H. , Nagy, A.-L. , Djerdjouri, B. , & Clichici, S. (2018). Precancerous ACF induction affects their regional distribution forsaking oxidative stress implication in 1, 2-dimethylhydrazine-induced colon carcinogenesis model. Inflammopharmacology, 26(2), 457–468. 10.1007/s10787-017-0377-5 28733896

[fsn3864-bib-0033] Nelson, K. , Stojanovska, L. , Vasiljevic, T. , & Mathai, M. (2013). Germinated grains: A superior whole grain functional food? Canadian Journal of Physiology and Pharmacology, 91(6), 429–441. 10.1139/cjpp-2012-0351 23746040

[fsn3864-bib-0034] Perez, S. , Talens-Visconti, R. , Rius-Perez, S. , Finamor, I. , & Sastre, J. (2017). Redox signaling in the gastrointestinal tract. Free Radical Biology and Medicine, 104, 75–103. 10.1016/j.freeradbiomed.2016.12.048 28062361

[fsn3864-bib-0035] Raman, M. , Ambalam, P. , Kondepudi, K. K. , Pithva, S. , Kothari, C. , Patel, A. T. , … Vyas, B. R. (2013). Potential of probiotics, prebiotics and synbiotics for management of colorectal cancer. Gut Microbes, 4(3), 181–192. 10.4161/gmic.23919 23511582 PMC3669163

[fsn3864-bib-0036] So, W. K. , Law, B. M. , Law, P. T. , & Chan, C. W. (2016). Current hypothesis for the relationship between dietary rice bran intake, the intestinal microbiota and colorectal cancer prevention. Nutrients, 8(9), 569 10.3390/nu8090569 PMC5037554 27649240

[fsn3864-bib-0037] Soltan Dallal, M. M. , Mojarrad, M. , Baghbani, F. , Raoofian, R. , Mardaneh, J. , & Salehipour, Z. (2015). Effects of probiotic *Lactobacillus acidophilus* and *Lactobacillus casei* on colorectal tumor cells activity (CaCo-2). Archives of Iranian Medicine (AIM), 18(3), 167–172. https://doi.org/0151803/AIM.006 25773690

[fsn3864-bib-0038] Torre, L. A. , Bray, F. , Siegel, R. L. , Ferlay, J. , Lortet-Tieulent, J. , & Jemal, A. (2015). Global cancer statistics, 2012. CA: A Cancer Journal for Clinicians, 65(2), 87–108. 10.3322/caac.21262 25651787

[fsn3864-bib-0039] Verma, A. , & Shukla, G. (2014). Synbiotic (*Lactobacillus rhamnosus* + *Lactobacillus acidophilus* + inulin) attenuates oxidative stress and colonic damage in 1, 2 dimethylhydrazine dihydrochloride-induced colon carcinogenesis in Sprague–Dawley rats: A long-term study. European Journal of Cancer Prevention, 23(6), 550–559. 10.1097/CEJ.0000000000000054 25025584

[fsn3864-bib-0040] Walia, S. , Kamal, R. , Dhawan, D. , & Kanwar, S. (2018). Chemoprevention by probiotics during 1, 2-dimethylhydrazine-induced colon carcinogenesis in rats. Digestive Diseases and Sciences, 63(4), 900–909. 10.1007/s10620-018-4949-z 29427224

[fsn3864-bib-0041] Zhao, B.-B. , Meng, J. , Zhang, Q.-X. , Kang, T.-T. , & Lu, R.-R. (2017). Protective effect of surface layer proteins isolated from four *Lactobacillus* strains on hydrogen-peroxide-induced HT-29 cells oxidative stress. International Journal of Biological Macromolecules, 102, 76–83. 10.1016/j.ijbiomac.2017.03.160 28366852

